# Identifying barriers to pediatric dental appointments: insights from a study at Prince Faisal bin Al-Hussein Hospital

**DOI:** 10.25122/jml-2024-0178

**Published:** 2024-08

**Authors:** Ahmad Mudar Khries, Razan Jameel Salaymeh

**Affiliations:** 1 Department of General Internal Medicine, Western Health and Social Care Trust, Northern Ireland, UK; 2 Department of Dental Medicine, Ministry of Health, Amman, Jordan

**Keywords:** Pediatric dentistry, non-attendance, children, dentistry, questionnaire, oral health

## Abstract

Ensuring good oral health is crucial for overall well-being. Missed appointments can negatively impact the quality of care and oral health outcomes, making it essential to identify the factors contributing to non-attendance. This study aimed to identify the factors associated with non-attendance at a pediatric dental clinic. The study used a cross-sectional design, which included a random sample (*n* = 265) of eligible pediatric patients under 12 years old, with data collected through questionnaires completed by their parents. Data were collected over 3 months, from November 2023 to January 2024, and analyzed using descriptive statistics and a chi-square test, with a confidence level of 95%. Results indicated that 76% of patients were school-age children, and 44% of children had missed their appointments. The critical barriers to attendance mentioned by parents were social and family commitments (15.2%), forgetfulness (11.2%), illness (8.6%), school commitments (6.9%), and fear of dental treatment (3.5%). The administrative barriers were due to staff miscommunications (10.3%), while non-attendance for unspecified reasons was 11.2%. Finally, the relationship between age, gender, and type of transportation with missed appointments was statistically significant (*P* < 0.05). This study highlights the significant rate of missed appointments and the factors contributing to non-attendance at pediatric dental clinics.

## INTRODUCTION

Dental appointments are crucial for maintaining oral health, encompassing discussions, examinations, and treatment procedures. Regular dental visits are essential because dental and oral diseases develop slowly and require periodic and regular check-ups to diagnose and detect dental problems [1]. Despite the importance of these visits, many patients seek treatment only when they feel moderate or severe pain, swelling, or infection [1,2]. Regular follow-up is vital to effectively monitor and manage dental conditions and complications [1-3].

Missed dental appointments disrupt continuity of care, especially for children, leading to wasted resources, interrupted workflow, and deteriorating oral health [1,4]. An appointment is missed if a patient fails to attend, cancel, or reschedule [5]. Lack of awareness and knowledge contribute to appointment cancellations and non-attendance [1]. If not addressed promptly, simple dental issues in children can escalate into severe problems, emphasizing the need for well-organized appointment systems [1,5]. Missed appointments significantly impact patients, the clinic's work, and the healthcare system [6]. Although dentists pay attention to scheduling appointments, providing their cards, recording the appointment date and time in each patient’s file, and reminding them of their appointments, dental clinics still have a high rate of missed appointments [7].

The pediatric dental clinic offers therapeutic and preventive care to preserve the oral health of pediatric patients. Regular dental appointments are important for early detection and treatment of oral health issues, and missed appointments lead to suboptimal dental care and poor oral health outcomes [8,9]. The consequences of missed appointments include complications from untreated conditions, wasted hospital resources, and underutilized staff [10,11]. Additionally, missed appointments can result in missed healthcare opportunities, leading to further complications [12]. This issue can also disrupt pediatric dentistry residency programs at teaching hospitals, where residents need to meet specific requirements and closely monitor some instances over time.

In Jordan, studies have highlighted issues related to oral health and caries in children under ten, linking them to factors such as poor oral hygiene, cultural attitudes, and attendance practices [13-15]. However, there is a lack of research evaluating the factors that lead to missed pediatric dental appointments and their impact on oral health in Jordanian children. Therefore, the main aim of this study was to evaluate and identify pediatric dentistry absenteeism from appointments in children under 12 years in Jordan.

This study aims to address this gap by evaluating the factors associated with missed pediatric dental appointments among children under 12 years old at Prince Faisal bin Al-Hussein Hospital in Al-Rusaifeh District, Jordan. It seeks to identify reasons for appointment cancellations, assess the relationship between demographic variables and non-attendance, and understand the implications of missed appointments on pediatric dental care. T

### Literature review

Numerous studies highlight the importance of understanding the barriers that prevent patients from attending dental clinic appointments. Identifying these barriers helps foster positive attitudes towards attending and adhering to appointments [1,16-17]. Therefore, comprehending the reasons and factors behind the high rate of missed appointments at dental clinics could contribute to more effective strategies for improving patient commitment to attending and thus reducing missed appointments [1,4,16,17]. Moreover, understanding the causes of missed appointments could inform interventions, such as a centralized electronic booking system or appointment reminders [18]. These interventions can enhance appointment adherence and optimize resource utilization, capacity, and dental training. Reducing missed appointments can improve pediatric oral health through better quality care [19]. A study by Sharma *et al*. [20] reported that a policy orientation addressing challenges related to missed appointments in dental clinics through tailored solutions based on perceived barriers helps patients keep appointments. In addition, previous literature reported high rates of missed appointments in pediatric dental clinics worldwide, with reports indicating rates as high as 80% [16,21-23]. Furthermore, recent studies have indicated that missing dental care appointments is affected by several factors, including socio-demographic characteristics, forgetfulness and confusion related to scheduling appointments, problems managing child behavior, and use of public insurance, as well as personal, financial, and structural barriers [24-27].

## MATERIAL AND METHODS

### Study design

This cross-sectional study used a descriptive and analytical approach, utilizing a structured questionnaire to gather relevant data. This study included records of pediatric patients who visited outpatient clinics and were treated at the pediatric dentistry clinic of Prince Faisal bin Al Hussein Hospital in Al-Rusaifeh, Jordan. The children were randomly and purposively selected.

### Data collection

Data was collected from participants’ records, including important social and demographic information such as date of birth, age, and gender, as well as dental records such as dental care appointments and visits to the children’s dental clinic at the hospital. Data and information were collected using a structured questionnaire (as a main source) over 3 months from November 2023 to January 2024. The period chosen took school vacation periods into account.

These surveys consisted of multiple-choice questions that covered the analytical facets of the subject matter as well as the goals and features of the research. In addition, the researchers relied on secondary sources, such as previously published theoretical and scientific works, such as books, peer-reviewed journals, university theses, and scientific research papers. Secondary sources are crucial in assisting researchers in evaluating major results and validating study hypotheses.

### Instrument

Parents and guardians of the participating children completed a pre-tested questionnaire that gathered comprehensive information on three main areas: child patient details, family background, and factors contributing to non-attendance. After obtaining informed consent, data were collected through direct interviews with the parents or guardians.

The questionnaire included:

**Patient information:** gender, age, nationality, dental insurance status, and medical condition of the child.

**Family information:** Details about household members, the number of children in the home, the parents’ occupation and education, and the means of transportation used to reach the pediatric dental clinic in the hospital.

**Factors for non-attendance:** These were categorized into: parent-related issues (e.g., social and family commitments, forgetfulness, parent or caregiver illness, work obligations, resolution of the dental issue); child-related issues (e.g., school commitment, fear of dental treatment, patient illness); administrative issues (e.g., long wait times, miscommunication, appointment gaps); and other unspecified reasons. The collected data were analyzed to identify the characteristics of patients/families who missed appointments and the main reasons for non-attendance.

### Validity

Experts pre-tested and reviewed the questionnaire to ensure validity, accuracy, and comprehensiveness. Feedback from these experts was used to refine and finalize the questionnaire.

### Study population and sample size

The study population included eligible pediatric patients who visited the clinic for dental treatment and had more than three appointments at the pediatric dental clinic (*n* = 265). Children under 12 years old were randomly selected. Data were collected from patient records over a specific period. The researchers contacted the parents or guardians of these pediatric patients to obtain informed consent for participation in the study. Eligible patients were identified by specifically selecting every third follow-up patient who met the inclusion criteria: under 12 years old and having attended more than three previous pediatric dental appointments. The only exclusion criterion was for patients who had attended fewer than three prior appointments.

### Statistical analysis

The data were analyzed and processed using the Statistical Package for the Social Sciences (SPSS) version 26 (IBM, Chicago, IL, USA). Descriptive statistics were used to analyze the demographic characteristics of the study sample (absolute frequencies and percentages for categorical variables), as well as means and standard deviations for continuous variables. The chi-square test assessed the relationships between missed appointments and gender, parents’ occupation, educational level, and type of transportation used. The significance level was set at *P* ≤ 0.05. To ensure the reliability of the questionnaire, the internal consistency was assessed using Cronbach's alpha, which yielded a reliability coefficient of 0.95, indicating excellent stability.

## RESULTS

The sociodemographic characteristics of the participants (*n* = 265) are presented in Table 1. The final sample comprised 265 pediatric patients aged between 2.5 and 12 years, with a mean age of 6.85 ± 1.7 years. Regarding gender, (57%) of participants were male participants, while 43% (*n* = 113) were female participants. A significant majority, 76% (*n* = 201), were school-age children. Also, 99.6% of the participants were Jordanian, and 85% (*n* = 225) had dental health insurance. Regarding medical conditions, 89% (*n* = 236) of participants had no significant medical history.

**Table 1:** Descriptive analysis of socio-demographic characteristics (*n* = 265)

Variables	Categorization	Frequency (*n*)	Percentage (%)
**Gender**	Male	152	57%
	Female	113	43%
**Age**	Pre-school	64	24%
	School	201	76%
**Nationality**	Jordanian	264	99.6%
	Non-Jordanian	1	0.4%
**Dental Insurance**	Yes	225	85%
	No	40	15%
**Medical condition**	Medically free	236	89%
	Positive medical history	29	11%

Table 2 presents the family-related information of the pediatric patients. Most (92%, *n* = 245) lived with both parents, and 73% (*n* = 193) were part of families with three or more children. Regarding the occupation and educational level of the fathers, 51% were employees, followed by those who were self-employed, 33% (*n* = 87), and 77% (*n* = 204) had a below-high school education. Regarding mothers, 89% (*n* = 237) were not employed outside the home (either not working or retired), and 74% had a below-high school education. Finally, 79% (*n* = 209) of the participants and their families relied primarily on public transportation to reach the hospital (Table 2).

**Table 2:** Descriptive analysis of family information (*n* = 265)

Variables	Categorization	Frequency (*n*)	Percentage (%)
**Child resides with**	Single parent or others	20	8%
	Two parents	245	92%
**Number of children**	One or two	72	27%
	Three and more	193	73%
**Occupation of father**	Employee	135	51%
	Not employed/retired	43	16%
	Self-employed	87	33%
**Occupation of mother**	Employee	23	9%
	Not employed/retired	237	89%
	Self-employed	5	2%
**Education of father**	Above high school	61	23%
	High school and below	204	77%
**Education of Mother**	Above high school	70	26%
	High school and below	195	74%
**Type of transport**	Car	39	15%
	Public transport	209	79%
	Walking	17	6%

Table 3 shows the statistical analysis results of the relationship between the age of the children, gender, parents’ occupation, parents’ educational level, type of transportation used, and missed pediatric dental clinic appointments. The results indicated no statistically significant associations between missed pediatric dental clinic appointments and parental occupation and the parent's educational level. However, significant correlations were identified between missed appointments and the child’s age, gender, and type of transportation used (*P* < 0.05).

**Table 3:** Association between studied variables and missed appointments

Variables	Categorization	Missed appointment		*P* value
		Yes (117), *n* (%)	No (148), *n* (%)	
**Gender**	Male	72 (62)	79 (53)	0.091*
	Female	45 (38)	69 (47)	
**Age**	Pre-school	24 (21)	40 (27)	0.0041*
	School	93 (79)	108 (73)	
**Occupation of father**	Employee	59 (50)	76 (51)	0.561
	Not employed/retired	18 (16)	25 (17)	
	Self-employed	40 (34)	47 (32)	
**Occupation of mother**	Employee	10 (9)	13 (9)	0.134
	Not employed/retired	105 (90)	132 (89)	
	Self-employed	2 (1)	3 (2)	
**Education of father**	Above high school	28 (24)	33 (22)	0.132
	Below high school	89 (76)	115 (78)	
**Education of Mother**	Above high school	31 (26)	39 (26)	0.129
	Below high school	86 (74)	109 (74)	
**Type of transport**	Car	15 (13)	24 (16)	0.002*
	Public transport	98 (84)	111 (75)	
	Walking	4 (3)	13 (9)	

* Significant at P < 0.05.

Table 4 shows the factors contributing to non-attendance at the pediatric dental clinic. The main reasons that parents or caregivers did not attend the pediatric patient clinic were social or family commitments (15.5%), followed by forgetfulness (11.2%), their illness (8.6%), work obligations (6%), and the resolution of dental issues (4.3%). At the same time, patients reported that the most important reasons for missing appointments were school commitments (6.9%), fear of dental treatment (3.5%), and sickness (12.1%). Regarding administrative reasons, the results showed that these reasons were long waiting times on the day of the appointment (5.2%), miscommunications between parents or caregivers and the caring staff (10.3%), and long gaps between follow-up appointments (5.2%). Other unspecified reasons (11.2%) included issues such as the need for insurance card renewal or treatment expenses for uninsured children.

**Table 4:** Factors associated with non-attendance at the pediatric dental clinic

Factors	Categorization	Frequency (*n*)	Percentage (%)
**Non-attendance due to parent or caregiver issues**	Social and family commitments	18	15.5%
	Forgetfulness	13	11.2%
	Parent/caregiver illness	10	8.6%
	Work obligations	7	6%
	Resolution of dental issue	5	4.3%
**Non-attendance due to patient issues**	School Commitment	8	6.9%
	Fear of dental treatment	4	3.5%
	Patient illness	14	12.1%
**Non-attendance due to administrative issues**	Long wait time on the day of appointment	6	5.2%
	Miscommunication between the parent/caregiver and the caring staff	12	10.3%
	Long gaps between follow-up appointments	6	5.2%
**Non-attendance due to nonspecific reasons**	The need for insurance card renewal	7	11.2%
	Treatment expense for an uninsured child	6	

## DISCUSSION

The current study aimed to determine the factors associated with non-attendance at a pediatric dental clinic, the reasons for canceling or missing appointments, and the relationship between some study variables and non-attendance. Our study revealed that 76% of participants were school-age children. This age group might face specific challenges impacting their dental appointment attendance. For example, school commitments and extended dental treatment times could contribute to missed appointments, as suggested by previous research [28]. Despite 85% of the participants having dental health insurance, the study noted a high rate of missed appointments (44%). This indicates that financial barriers alone do not fully explain non-attendance. This result is consistent with some previous studies [1,24,29]. Moreover, other studies have found that practical obstacles like transportation, work schedules, and competing family obligations can impair appointment attendance [30]. The results showed an association between the age of the pediatric patient, gender, the type of transportation used, and missed pediatric dental clinic appointments in the current study. This result is consistent with a study conducted by Bhatia *et al*. [5].

Our study found that the low educational level of parents in this predominantly Jordanian sample may reduce their oral health knowledge and make navigating children's dental care regimens more difficult [31]. This result is consistent with studies conducted in Germany, which confirmed that the importance of parents' educational level emerged in the relationship with the risk of tooth decay in children [32,33]. Higher parental education levels have been linked to better oral health outcomes, and fewer missed appointments [34,35].

The correlation between the type of transportation and missed appointments was statistically significant, which is predictable given that most participants rely on cramped and unreliable public transport, and the existing infrastructure may not sufficiently facilitate access.

The study focused on obstacles to not attending and missing appointments at pediatric dental clinics, including barriers related to parents or caregivers, pediatric patients, and administrative reasons. The results showed that the most important barriers related to the absence of parents or caregivers were social or family obligations, forgetfulness, and illness. Parents' or caregivers' social or family commitments and forgetfulness were among the most common reasons for non-attendance. This may be because the scheduling period and date of booking appointments are long, making it difficult for parents to remember them. These results are consistent with many previous studies, which confirmed that the most common reasons for the increase in missed appointments are forgetfulness and parental preoccupation [5,36,37]. Parents or caregivers can reduce missed appointment rates in pediatric dental clinics using appointment reminder cards and mobile phones [5]. The results indicated that 4% of missed appointments were due to resolving dental issues, which holds importance. Pediatric dentistry treatment plans for all patients include preventive measures as a central component. Therefore, parents or caregivers should be educated about the importance of attending appointments even if the dental issue has been resolved, emphasizing that regular check-ups are crucial for maintaining oral health. Aside from external barriers, intrinsic factors associated with pediatric patients, such as their fear of dental treatments, can directly affect their willingness to attend. This may be because severe anxiety and traumatic experiences lead to avoidance of dental care at all ages. Pediatric dentistry can benefit from approaches that mitigate these fears, including advanced anesthetic techniques and child-friendly interventions [38]. Administrative issues were a notable factor in non-attendance, with 11.2% of missed appointments attributed to communication and office operational problems. Practical strategies to address these barriers include developing automated appointment reminders, clarifying cancellation and no-show policies, reducing wait times, and shortening the gaps between follow-ups [39]. Furthermore, clear and good communication of behavioral expectations and best practices increases appointment adherence and engagement in treatment [40].

Overall, our results provide a glimpse into the barriers and problems that lead to a high rate of missed appointments and, thus, an increase in oral health problems in children. Therefore, overcoming barriers at the parent, patient, health care provider, and system-specific levels in pediatric dental clinics can significantly improve attendance and long-term oral health outcomes for children [41]. Researchers should focus their future studies on creative solutions that require further exploration, whether through policy reforms, dental practice innovations such as school-linked programs and mobile dentistry, or grassroots oral health literacy campaigns designed for vulnerable families [42]. Also, emphasis should be placed on various methods that reduce missed appointments at pediatric dental clinics and encourage patients to attend, thus enhancing oral health and treatment results. It is also possible to rely on modern technologies to help follow up on the case. An example of this is the use of applications that can be installed on smartphones, through which one can monitor the patient and know the condition related to oral health [43, 44]. Sometimes, a child may become anxious and feel fearful of loud-sounding devices. To avoid these sounds, some companies have developed special devices to prevent or control noise in the dental clinic, such as Quieton and Alltalk [45]. Another study indicated calming music reduces anxiety and improves patients’ health [46]. Technologies such as the SleeperOne S4, which controls the amount of local anesthesia, can help alleviate the fear of needles and improve the comfort of dental procedures [ 47]

Although our current study provides a valuable perspective, we faced some limitations, including the fact that the study design was limited to a sample in a specific region in Jordan, which limits the possibility of generalizing on a broader scale. Additionally, response bias in self-reported data may influence the findings. Future research should expand to include diverse regions and socioeconomic backgrounds to provide a more comprehensive understanding of the barriers to dental appointment adherence. Integrating dental record data with survey results could also enhance the validity of the findings [48].

## CONCLUSION

The study identified key factors contributing to non-attendance at pediatric dental clinics, offering valuable insights into the obstacles preventing children and their parents from seeking dental care in Jordan. The results indicated that the obstacles included aspects related to parents, pediatric patients, and health system administration. In addition, the relationship between non-attendance and study variables was determined, and it was found that non-attendance at pediatric dental clinic appointments was significantly related to the age and gender of the patients, as well as the type of transportation. Additionally, this study sheds light on the obstacles causing many missed appointments, leading to increased oral health issues in children. We recommend implementing grassroots oral health campaigns targeting vulnerable families. Overcoming practical obstacles, addressing fear of dental treatment, and improving appointment coordination can lead to significant improvements.

## References

[1] Anagha KA, Megha M, Karuveettil V, Kumar SV (2024). Perceptions of barriers towards dental appointment keeping among patients of a tertiary care setting: A mixed method exploration. J Oral Biol Craniofac Res.

[2] George AC, Hoshing A, Joshi NV (2007). A study of the reasons for irregular dental attendance in a private dental college in a rural setup. Indian J Dent Res.

[3] Chariatte V, Berchtold A, Akré C, Michaud PA, Suris JC (2008). Missed appointments in an outpatient clinic for adolescents, an approach to predict the risk of missing. J Adolesc Health.

[4] Desai RV, Srivastava BK, Eshwar S, Jain V (2018). A cross-sectional study to assess the dental appointment attendance, reflecting the experiences, anticipations and behavioural intentions among 18-25 year olds. Int J Appl Dent Sci.

[5] Bhatia R, Vora EC, Panda A (2018). Pediatric dental appointments no-show: rates and reasons. Int J Clin Pediatr Dent.

[6] Ismail AI, Saeed MH, AlSilwadi FM (2011). Missed dental appointments in the United Arab Emirates. J Int Dent Med Res.

[7] Bellucci E, Dharmasena L, Nguyen L, Calache H (2017). The effectiveness of SMS Reminders and the impact of patient characteristics on missed appointments in a public dental outpatient clinic. Australas J Inf Syst.

[8] Akter S, Doran F, Avila C, Nancarrow S (2014). A qualitative study of staff perspectives of patient non-attendance in a regional primary healthcare setting. Aust J Med.

[9] Gomes MAG, Abreu MHNG, Ferreira FM, Fraiz FC, Menezes JVNB (2019). No-shows at public secondary dental care for pediatric patients: a cross-sectional study in a large Brazilian city. Cien Saude Colet.

[10] Brewster S, Bartholomew J, Holt RI, Price H (2020). Non-attendance at diabetes outpatient appointments: a systematic review. Diabet Med.

[11] Guedes R, Leite I, Baptista A (2014). Dermatology missed appointments: an analysis of outpatient non-attendance in a general hospital's population. Int J Dermatol.

[12] Kirby J, Harris JC (2019). Development and evaluation of a 'was not brought' pathway: a team approach to managing children's missed dental appointments. Br Dent J.

[13] Aljafari A, ElKarmi R, Nasser O, Atef A, Hosey MT (2022). Oral health status and practices of 6-to 7-year-old children in Amman, Jordan: a cross-sectional study. BMC Oral Health.

[14] Al Zoubi L, Schmoeckel J, Mustafa Ali M, Splieth CH (2021). Parental acceptance of advanced behaviour management techniques in paediatric dentistry in families with different cultural background. Eur Arch Paediatr Dent.

[15] Hammouri EH, Mustafa AT, Jaradat TF, Ghozlan MM, Bani Salman MY, Ersheidat AA (2024). Exploring Jordanian children and parents' awareness, behavior, and perception of pediatric oral health. BMC Oral Health.

[16] Souza-Oliveira AC, Paschoal MA, Alvarenga-Brant R, Martins CC (2023). Frequency of missing data in clinical records in pediatric dentistry: a descriptive study. J Clin Pediatr Dent.

[17] Karuveettil V (2023). Perceptions of barriers towards dental appointment keeping among patients of a tertiary care setting: a mixed method exploration. Popul Med.

[18] Masoud T, Shah A, Joomun S (2017). Reducing DNA rates and increasing positive contacts in an outpatient chronic fatigue service. BMJ Qual Improv Rep.

[19] da Cunha IP, de Lacerda VR, da Silveira Gaspar G, de Lucena EHG, Mialhe FL, de Goes PSA (2022). Factors associated with the absence of Brazilians in specialized dental centers. BMC Oral Health.

[20] Sharma S, Cruise A, Salamasi H, Narula A (2014). Non-attendance at the ent outpatient clinic. Bull R Coll Surg Engl.

[21] Kheirkhah P, Feng Q, Travis LM, Tavakoli-Tabasi S, Sharafkhaneh A (2016). Prevalence, predictors and economic consequences of no-shows. BMC Health Serv Res.

[22] Aggarwal A, Davies J, Sullivan R (2016). “Nudge” and the epidemic of missed appointments: can behavioural policies provide a solution for missed appointments in the health service?. J Health Organ Manag.

[23] Ogordi PU, Edetanlen EB (2023). Prevalence and risk factors of missed appointment among paediatric patients after minor oral surgical procedures in a tertiary hospital in Southern Nigeria. S Afr Dent J.

[24] Goldman K, Aldosari MA, Discepolo K (2022). Missed Dental Care Appointments in an Urban Safety Net Hospital. J Calif Dent Assoc.

[25] Khalid G, Metzner F, Pawils S (2022). Prevalence of dental neglect and associated risk factors in children and adolescents—a systematic review. Int J Paediatr Dent.

[26] Elkhodary HM, Bagher SM, Sabbagh HJ, Almushayt A, Almalik M, Baghlaf K (2022). Factors relating to failure rates of dental procedures in children following comprehensive dental treatments under general anesthesia: A 2-year retrospective study. Niger J Clin Pract.

[27] Nowak A, Dooley D, Royston L, Rust S, Hoffman J, Chen D (2018). Predictive model for caries risk based on determinants of health available to primary care providers. Am Acad Pediatr Dent.

[28] Bhagat D, Khan MH, Uddin MA (2014). Barriers for parental failure in bringing their children to the dental clinic—A questionnaire-based study in Indian population. Indian J Med Sci.

[29] Tandon S, Duhan R, Sharma M, Vasudeva S (2016). Between the cup and the lip: missed dental appointments. J Clin Diagn Res.

[30] Chapman KA, Machado SS, van der Merwe K, Bryson A, Smith D (2022). Exploring Primary Care Non-Attendance: A Study of Low-Income Patients. J Prim Care Community Health.

[31] Lee W, Kim SJ, Albert JM, Nelson S (2014). Community factors predicting dental care utilization among older adults. J Am Dent Assoc.

[32] Deutsche Arbeitsgemeinschaft für Jugendzahnpflege (DAJ) editor (2017). Epidemiologische Begleituntersuchungen zur Gruppenprophylaxe 2016. Deutsche Arbeitsgemeinschaft für Jugendzahnpflege.

[33] Schmoeckel J, Santamaría RM, Splieth CH (2015). Long-term caries development in schoolchildren and the role of educational status. Quintessence Int.

[34] Cianetti S, Lombardo G, Lupatelli E, Rossi G, Abraha I, Pagano S (2017). Dental caries, parents educational level, family income and dental service attendance among children in Italy. Eur J Paediatr Dent.

[35] Rajab LD, Petersen PE, Baqain Z, Bakaeen G (2014). Oral health status among 6-and 12-year-old Jordanian schoolchildren. Oral Health Prev Dent.

[36] AlSadhan SA (2013). Frequency of missed and cancelled appointments in King Saud University orthodontic clinic. King Saud Univ J Dent Sci.

[37] Lacy NL, Paulman A, Reuter MD, Lovejoy B (2004). Why we don’t come: patient perceptions on no-shows. Ann Fam Med.

[38] Remi RV, Anantharaj A, Praveen P, Prathibha RS, Sudhir R (2023). Advances in pediatric dentistry: new approaches to pain control and anxiety reduction in children-a narrative review. J Dent Anesth Pain Med.

[39] LaGanga LR, Lawrence SR (2007). Clinic overbooking to improve patient access and increase provider productivity. Decis Sci.

[40] Curado C, Henriques PL, Jerónimo HM, Azevedo J (2022). The contribution of communication to employee satisfaction in service firms: A causal configurational analysis. Vision.

[41] Lai YYL, Downs JA, Wong K, Zafar S, Walsh LJ, Leonard HM (2022). Enablers and barriers in dental attendance in Rett syndrome: an international observational study. Spec Care Dentist.

[42] Mofidi M, Rozier RG, King RS (2002). Problems with access to dental care for Medicaid-insured children: what caregivers think. Am J Public Health.

[43] Pascadopoli M, Zampetti P, Nardi MG, Pellegrini M, Scribante A (2023). Smartphone applications in dentistry: a scoping review. Dent J.

[44] Timmers T, Janssen L, Kool RB, Kremer JA (2020). Educating patients by providing timely information using smartphone and tablet apps: systematic review. J Med Internet Res.

[45] Kim IH, Cho H, Song JS, Park W, Shin Y, Lee KE (2022). Assessment of real-time active noise control devices in dental treatment conditions. Int J Environ Res Public Health.

[46] Tziovara P, Antoniadou C, Antoniadou M (2024). Patients’ perceptions of sound and noise dimensions in the dental clinic soundscape. Appl Sci.

[47] Vitale MC, Gallo S, Pascadopoli M, Alcozer R, Ciuffreda C, Scribante A (2023). Local anesthesia with SleeperOne S4 computerized device vs traditional syringe and perceived pain in pediatric patients: a randomized clinical trial. J Clin Pediatr Dent.

[48] Agrawal AA, Prakash N, Almagbol M, Alobaid M, Alqarni A, Altamni H (2023). Synoptic review on existing and potential sources for bias in dental research methodology with methods on their prevention and remedies. World J Methodol.

